# Health and Academic Performance With Happy Children: A Controlled Longitudinal Study Based on the HOPP Project

**DOI:** 10.3389/fcvm.2022.820827

**Published:** 2022-06-03

**Authors:** Nandu Goswami, Dominique Hansen, Goran Gumze, Bianca Brix, Karin Schmid-Zalaudek, Per Morten Fredriksen

**Affiliations:** ^1^Physiology Division, Otto Loewi Research Center, Medical University of Graz, Graz, Austria; ^2^Health Sciences, Alma Mater Europaea, Maribor, Slovenia; ^3^Mohammed Bin Rashid University of Medicine and Health Sciences, Dubai Healthcare City, Dubai, United Arab Emirates; ^4^REVAL/BIOMED, Faculty of Rehabilitation Sciences, Hasselt/Heart Centre Hasselt, Jessa Hospital, Hasselt University, Hasselt, Belgium; ^5^Health Sciences, Alma Mater Europaea ECM, Maribor, Slovenia; ^6^Faculty of Health and Social Science, Inland Norway University of Applied Sciences, Hamar, Norway; ^7^School of Health Sciences, Kristiania University College, Oslo, Norway

**Keywords:** school-based physical activity, school intervention, elementary school children, physical fitness, academic achievement, children's health

## Abstract

**Background:**

Overweight/obesity in children and adolescents, largely arising due to increased food intake and reduced physical activity, is a major health concern. Physical activity (PA) integrated into learning has been shown to not only lead to improved health outcomes and wellbeing but also positively affect academic performance. The Health and Academic Performance with Happy Children (HAPHC) project aims at enhancing health and academic performance in elementary school children *via* implementation of a daily unit of Physical Activity Across the Curriculum (PAAC), which is carried out within the school setting. In this project, PA as an integrated part of learning will be evaluated and the learning material adapted for a large scale implementation across several European countries.

**Methods:**

In three European countries (Austria, Slovenia, and Belgium), 12 primary schools in total will be recruited to act as either intervention or control school in a large intervention study, which applies the PAAC pedagogy during lectures. It is estimated that, at least 3,000+ children across the three countries will be recruited in this study. All teachers of intervention schools will receive training and materials/teaching equipment that will allow them to integrate a daily PA unit of 45 min over 3 years across the curriculum. In response to the daily PA intervention, the following primary outcomes will be assessed: changes in health related physiological factors, academic achievement, psycho-social aspects and wellbeing.

**Impact of Project:**

The HAPHC project aims at promoting public health by increasing PA at an early age within the school setting and therewith preventing the increasing risk of non-communicable diseases across Europe. HAPHC project aims to develop knowledge and materials, which will ensure that the PAAC can be scalable to other European countries.

**Trial Registration Number:**

ClinicalTrials.gov, identifier: NCT04956003.

## Background

All countries in the world are faced with a tremendous challenge of overweight/obesity and physical inactivity. Despite repeated efforts, the problem has spread over the globe in pandemic proportions. In particular, the numbers of overweight/obesity in children and young adolescents in developing countries are alarming (1, 2). European countries are also registering a clear trend toward less physical activity (PA) in children and adolescents (3) and with it, a rising prevalence rate of overweight/obesity, diabetes and related mental and physical health issues. The current COVID-19 pandemic has further worsened the prevalence of these cardio-metabolic disorders (4, 5).

For more than 10 years, the WHO European Childhood Obesity Surveillance Initiative (COSI) has systematically registered data from primary school children in more than 40 member states. Prevalence rates of overweight and obesity vary widely from 2–5% up to 28–30% (6). In most countries, a tendency toward further increases in overweight and obesity are seen (6). Overweight and obesity in children and adolescents has been shown to persist into adulthood (7, 8), where it can constitute as a major risk factor for premature onset of cardio-metabolic diseases (6, 9, 10). If unchecked, the harmful health consequences resulting from overweight and obesity could potentially overwhelm the health care systems worldwide in the next few years (11, 12). There is need for a global public health effort to halt the increasing prevalence of overweight/obesity and associated non-communicable diseases (NCD's), especially among children.

Healthy nutrition and PA are the key lifestyle factors in the prevention of overweight and obesity (13). Aside their positive effects on cardiometabolic health, PA positively affects academic performance (14–17). PA has been shown to increase blood oxygen flow to the brain, elevate norephinephrine and endorphin levels, and, consequently, reduce stress and improve the mood (8). In addition, PA has been shown to lead to changes at the cellular level, which includes its role in support of synaptic plasticity [for a detailed review see (8)]. The positive effects of PA in children are not short term but also long term. The long-term beneficial effects of PA include reduction in the risk of cardiovascular disease (18, 19) and improvements in mental health, especially in adult life (20–22). Accordingly, at least 60 min of moderate to vigorous-intensity PA (MVPA) daily are recommenden by the WHO (10, 23). Intensive aerobe physical activities to strengthen muscle and bones should be performed at least 3 days per week as these exercises have been reported to have positive effects on blood pressure, blood glucose and lipid parameters, bone health, physical fitness, mental health as well as academic performance (17, 24).

About one third of a child's life is spent at school, and about 8–10 h 5 days a week this time is spent mainly sedentary attending lessons (3, 25, 26) with a strong trend toward decreasing PA with increasing age. Furthermore, many children lack the possibility and/or stimulation to PA at home (15, 27). Therefore, the school not only has a major responsibility to stimulate and promote healthy living in children, but also opens a wide time window to continuously and consequently integrate PA into children's everyday life (28). Several school-based PA interventions have been sucseccfully implemented in school-children of different age (17, 29–37). Results of the MOVI-Kids study for example indicated increased cardiorespiratory fitness and muscular strength and velocity after implementation of three 60 min PA sessions per week in 4–7 year old children after only a couple of months (36), and the Sogndal school-intervention study showed a reduction in a cardiovascular risk cluster score with a 60 min teacher controlled daily PA in elementary school children after 2 years (31, 35). Aside projects at site, several “online” intervention programs—providing materials, information and evaluation—similarly indicate increased PA and sustained improvement of cardiorespiratory fitness (37–39) and students' physical self-worth (32, 34, 40), and represent an alternative approach in unfavorable times such as pandemic related school lock-downs (41). One of those international programs is the KaziBantu project of the UNESCO Chair on Physical Activity and Health in Educational Settings, a school-based intervention program, aiming to improve physical education and healthy active living of school children and teachers in low-resource settings in South Africa (42, 43). Although “online” programs are well-received and offer a the possibility to implement PA at large scale and to low cost, continued project monitoring is needed to warrant the quality of implementation (38, 44, 45).

Including a health related dimension into teaching, not only as an oral/verbal transfer of information, but as an active part of a health promoting program for children seems the obvious next step against existing and future health problems. PA during school is, however, a trade-off between the time spent in PA to achieve positive effects on concentration, memory and class-room behavior that improves the educational value of each hour spent learning, and the decreased time spent in learning caused by more time spent in PA (14). To modify this trade-off, attempts to integrate physical education in the regular curriculum, with the aim of attaining the value of physical education without loosing time to achieve this goal, have been made. These approaches are commonly referred to as PAAC (Physical Activity Across the Curriculum) or active learning (15, 23, 46–48). A systematic review of studies including PAAC provides clear evidence that programs increasing PA not only result in improved physical fitness but also in improved academic outcomes (17, 29, 47, 48).

One of the largest national programs, with regard to sample size and duration, is the ongoing 7-year longitudinal controlled trial “the Health Oriented Pedagogical Project” (HOPP), performed in the south-east part of Norway. HOPP follows more than 2,300 elementary school children, the youngest 6 years of age at time of inclusion (2015), to the end of elementary school in 2021 (49–57). Based on the experience and preliminary results from the HOPP, transfer of knowledge as to how to educate children in a more joyful and intuitive way is fundamental. In contrast to other international campaigns, PAAC together with other health education relevant aspects, is continuously performed since 2015 as an integrated part of teaching and will provide insight into the long-term effects of increased PA on health, academic perfornance and wellbeing. Based on the knowledge and materials already successfully applied in the HOPP, the dissemination to other European countries is eminent.

## Problem Statement

Although several programs for active learning have been developed, PAAC has not yet been consolidated integrating current knowledge as well as data from the HOPP study. To achieve the best possibilities in each individual considering a healthy and productive life, as well as for society to have healthy, hardworking citizens that contribute to society-building activities, it is fundamental to promote health at an early age. Moreover, there is a need to change sedentary learning and teaching practices, providing an optimal learning and health promoting environment, to a more health enhancing pedagogy. The “Health and Academic Performance with Happy Children” (HAPHC) project allows investigation of different factors related to learning as well as health outcomes resulting from enhanced PA. Mental health among children and adolescents has for good reason been set high on the political agenda through its important role for future public health and productivity. PA has shown to have an important association with mental health outcomes such as quality of life (58), wellbeing and physical self-worth (40). Along with the implementation of PAAC, the HAPHC-study aims to establish an educational program and practice for teachers at university level to incorporate PA into the curriculum by providing methods and materials for teaching. Validation of the health and educational effects by application of consistent methods across the participating European schools, and collaboration with similar international programs is required.

## Aims and Objectives

The overall aim of the longitudinal controlled HAPHC-study is to enhance health and academic performance by use of PAAC and therewith support children's quality of life and wellbeing. By application of the same well-established methods and materials in three European countries, evaluation of effects is performable at large-scale, a question that was left largely unexplored in many previously reported studies (29). Based on the findings of the HAPHC project, key health risk factors should be identified and practical knowledge for the implementation is expected, to provide adaptation for the transfer to all European countries in near future.

Specific research questions of the HAPHC project are with regard to efficiency:

1 Physical activity and health related outcomes: How/does a daily unit of MVPA integrated into learning change physical fitness, PA during leisure time and cardio-metabolic health of elementary school children?
2 Academic achievement: How/does a PAAC pedagogy change academic achievement, in particular with regard to main topics (maths, language) and executive function?
3 Mental health and quality of life: How/does a daily unit of MVPA integrated into learning change a child's mental state (anxiety, depression), quality of life and well being?

Secondary research objectives of the HAPHC project are with regard to pedagogy, feasabiliy and dissemination:

1 Development and adaptation of teaching equipment/material for PAAC in elementary schools.
2 Dissemination of knowledge and large-scale pan-European transfer.
3 Integration into the elementary school's curriculum and into teacher's training/education at university level.
4 Promotion of public health and prevention of NCDs.

This research further aims at promoting public health within increased PA, healthy diet and prevention of NCD's among children throughout elementary schools. The focus is on the following thematic areas: (i) learning and cognition, (ii) nutrition and growth, including obesity, (iii) PA and health, (iv) mental health and quality of life. Children of both sexes will perform similar PAAC learning throughout the project. This will empower all girls and boys from different socioeconomic, cultural and health status settings and contexts to perform PA while learning.

The specific objectives of HAPHC are:

*1 Encourage PA helping children from all socioeconomic backgrounds and cultures*. Several projects have been conducted in Europe (ASK, HOPP, STOP) (46, 59), USA (SWITCH, PAAC) (60, 61), Australia (TransformUs) (62) and South-Africa (KaziBantu) (63). The major objections against of these projects are too short interventions or too small sample sizes, or both. HAPHC will be based on the experience from the HOPP-study in Norway (46), and the challenge is to incorporate a pedagogy independent of culture which may be used across Europe. PAAC have the strength to reach all socioeconomic layers of a society and hence may have a short and long term effect on public health and education level. There are several knowledge gaps the current study fills and aims to expand the theoretical foundation of a PAAC program through comparative studies across Europe, integrating theoretical perspectives of other PAAC programs. Furthermore, the study aims to expand the empirical foundation of PAAC program by collaborating with international R&D institutions that are setting up similar empirical studies in different parts of Europe (15, 60).
2 Create an alliance with schools and teachers. To enhance academic performance, the study aims to develop international/intercultural transferable toolkits on active learning—which will lead to better educational results. This includes for instance, integrating PA in subjects like mathematics and change the teaching approaches.
3 Ensure better public health for now and in the future. Reducing lifestyle related diseases in the adult community by preventing an early-onset in childhood is the most efficient way of healthcare that European countries can provide.
4 Improve health-related economy by reducing the upcoming tsunami of health problems related to NCD's. Key knowledge needs and gaps that this project addresses are:

a How should the different curricula best be adjusted to incorporate PA?
b How should generic approaches be tailored to the different school settings?
c How can we ensure that the ongoing initiatives have positive effects on education outcomes?
d How to get support from subjects which examine causal mechanisms (e.g., medicine, psychology)?
e How to facilitate cooperation between researchers and practitioners?
f How to facilitate cooperation across cities participating in this project? The novelty of the present study is the pan-European focus, allowing involvement of school-aged children across Europe, starting with Austria, Belgium and Slovenia.
g How can PAAC be transferred to different cultural and social settings? The experience from HOPP shows that differences in culture between different parts of Norway requires different approaches. The innovative component can be seen as well in adjustment of the practice for—and transfer of the practice into different cultural and social settings. Especially as not all societies have the same attitude toward the perception of physical fitness, health and learning methods. Similarly, there is also an attitude toward PA that needs to be considered.

## Methods/Design

HAPHC will focus on a new pedagogical approach, PAAC, on a large-scale implementation in elementary schools across three European countries: Austria, Slovenia and Belgium.

### Training Teachers in Implementing the PAAC Program

This study specifically targets knowledge alliance in the area of development of pedagogical skills for teachers to implement PAAC in their schools. Replacing sedentary theoretical activities with PAAC, using schools as an intervention arena reaches all socioeconomic layers with the potential of reducing inequality in health and education level. All partners in the enterprise will be involved in establishing PAAC in their countries, and at several elementary schools of which some are partners of the consortium as well as at a teachers training school (Austria), materials for implementation of PAAC will be developed.

Teachers from each participating school will receive education, by experts from Norway, in how to implement PAAC. These training courses will include lectures and practice in elementary schools in Horten municipality in Norway, where HOPP has been conducted. In addition, teachers will be instructed in how to develop equipment and material for PAAC, and discuss ideas as how to adapt the pedagogy to their own culture and curriculum on different grades. Therewith, the schools and teachers partly take over the responsibility for their own pedagogy and will be more motivated to implement PAAC.

Aside the support of providing developed materials, teachers are asked to record the number of PAAC lessons they performed weekly. The materials provide several tasks to integrate physical activity in the classrooms. The tasks require the children's physical and mental efforts. They include elaboration of new material as well as repetition of already achieved material and cover the following subjects: mathematics, maternal language, English and science. Teachers will continue to redesign the instructional approach and learning materials during the implementation of active learning to optimize them based on their local curricula and other specific requirements. Teachers and principals from participating schools will also play an important role in transferring information to parents and pupils. Furthermore, the teachers and principals will present the aims and ideas to the professional community on a political level.

The current project builds upon the HOPP project, and by active collaboration between the consortium members, excellent teaching will be promoted and skills developed to encourage PA in the lectures (46). The intervention will be performed by teachers of the participating schools for at least 3 years. Involved researchers will support development and adaptation of methodology, and will perform measurements to assess health and academic effects. Although the design does not allow for randomized assignment of participants, the natural environment in the sense of a field study with familiar teachers performing a new pedagogy (PAAC) as well as the large number of participants will—together with a case-control methodology—provide a stronger knowledge platform than smaller-scale RCT studies.

### Population and Sample Size

Schools will be invited to participate either as intervention or control school. In the attempt to be most inclusive, all children of grades 2 and 3 of the participating schools are allowed to take part. In intervention schools, all children will be taught with PAAC, though only children whose parents/legal representatives gave written informed consent will be included in the measurements of physical fitness, health parameters, cognitive/mental and social abilities. Children of both sexes, different socio-economic backgrounds and cultural contexts, and from urban as well rural areas will be enrolled. No child will a priori be excluded, neither from physically active learning nor from the measurements performed. However, any pre-existing disability or illness will be recorded and considered when validating the results for the children. Furthermore, each child may deny participation at any time in the course of the study or for a certain measurement.

In each country, aged-matched children of a different school will be included as the control group. Healthy children of both sexes, aged 7–9 years (in grade 2–3), will be tested at the same time intervals as children from the intervention schools. Children in the control group will not receive active learning throughout the duration of the project, but will be taught according to the usual didactics.

Assuming that the prevalence of NCDs varies across schools, following a normal distribution with a mean value of 3% and a SD of 2%, a prevalence rate of about 5% would be expected. The power calculation for the intervention study is based on the change in a quantitative outcome variable from baseline to follow-up. Annually analyses between intervention and control schools in numerous variables, the sample size for a parallel group design is calculated using *n* = 2 · (σ / Δ) 2 · k, where n represents the number of participants in each group, σ (sigma) the expected standard deviation of the observations (which is assumed to be equal for all groups), Δ (delta) gives the relevant difference between observations, and k is the constant in this study, chosen based on the common values by a double-sided *t*-test set at 5% and with test strength 80% (this corresponds to a *k* of 7.9). Based on these calculations we have determined the sample size to be adequate for all variables for all ages, also when divided by sex (Table 1).

**Table 1:** Based on the various tests, the following sample sizes for each group (intervention and control schools) were calculated as adequate for analyses, with a power of 80% and alpha of 5%.

	**Variables**	**Methods**	**Number of children in each group/total**
Anthropometry	Weight, height, IsoBMI, fat-, fat-free and muscle-mass, WHtR	Measuring tape, body composition analyzer	34^*^/68 and 64^**^/128
Physical activity level	MVPA 60 min/day, VPA (min/day) and inactivity (min/day)	24-h activity monitor (Actigraph) for 1 week	126^***^/252
General physical capacity (fitness)	Strength test (kg) and Andersen Running Test	Jamar Handgrip	73^*^/146
Blood values	HDL and total cholesterol, Glycated hemoglobin (HbA1c), hemoglobin, hematocrit, ferritin, iron, and red blood cell (RBC) count	Blood analysis	126^***^/252
Quality of Life and SDQ?	LQ_0−28_	ILC questionnaire, parental and child response	34^*^/68
Diet	Intake of fat, sugar, fruit/vegetables and confectionary in diet	Questionnaire	73^*^/146
Cognitive ability	Executive functions	Eriksen flanker test Stroop test	24^*^/48
Behavioral screening	Mapping of emotions, body image and friendship	BUS-Test	85^*^/170
Academic performance	Score in national test for English, Mathematics and native language	Local and national academic tests	34^*^/68

*Statistical program: Systat 13, Power analyses*.

* *one-sample t-test*,

** *two-sample t-test*,

*** *Z-score*.

In the present study we will recruit ~3,000 + pupils in three countries. As can be seen in Table 2, the number of pupils recruited are sufficient for analyzing the questions established in Table 1.

**Table 2:** Estimated sample size for HAPHC.

	**Intervention schools**	**Control schools**	**Total**
Austria	558	198	756
Slovenia	936	225	1,161
Belgium	798	398	1,196
Total *n* of children	2,292	821	3,113

### Statistical Protocol

All children will be measured regarding the variables of interest (Table 1) before the implementation of PAAC starts at each intervention school as well as in the control schools (baseline). At the end of the first school year with PAAC in intervention schools, all children will be measured/tested again (1st follow-up). The same measurements/tests will be performed after the 2nd and 3rd year of implementation in all schools.

All data collected will be explored with regard to their distribution and potential outliers before being further analyzed. Main focus of the analyses will be the evaluation of changes in parameters of interest (physical fitness, anthropometry, quality of life, cognition etc.) from baseline to follow-up(s), depending on the intervention. Thus, comparing if the magnitude of change differs between children from intervention schools compared to children of the control schools. Depending on the number of variables and time-points to be compared in one analysis, dependent *t*-tests, repeated measures ANOVA and/or ANCOVA will be performed. Accordingly, a higher increase of, e.g., physical fitness in children of the intervention schools compared to children of a control school is expected.

In case of violations against the assumptions of parametric tests, either data transformations or application of non-parametric tests will be performed.

### Ethical Considerations

After detailed information on the project aims and measurements performed on children, which will be provided within teacher-parents evenings by researchers and local teachers, written informed consent forms signed by the parents or legal representatives will be collected. Children without permission to participate will only take part in the pedagogy, which will be performed in all classes of each intervention school. Furthermore, children will be given a detailed explanation and demonstration of the measurements before their consent forms will be collected, and will be given the chance to deny a certain measure or test at any time in the course of the study. All data will be anonymized and measures will be conducted according to the principles stated in the Declaration of Helsinki (2013). Data on physical characteristics such as height, weight, strength etc. will not be verbally communicated in the presence of other children.

## Discussion

The HAPHC-project will provide a large-scale dataset on how PA successfully can be integrated into curriculums in different European countries and how children will benefit from PAAC. By active collaborations between the consortium members, HAPHC will provide excellent teaching skills, based on the idea of a pan-European intervention to encourage PA during teaching to reach all socioeconomic, cultural and health layers of the child population. Creation of an open platform where school authorities can collaborate on educational content, validation and general experience sharing will be established. This virtual toolbox will allow coordination of activities across countries and school programs. Currently, few schools are using the PAAC approach, but this study aims to achieve an increase in such educational programs in Europe.

The main target groups in this study are children in primary schools across Europe. The prevention of NCDs has top priority in all countries across Europe, and the health benefits along with improved academic performance are at the center of the present intervention. HAPHC will potentially prevent NCDs and reduce cardiometabolic risk factors in a large child population, and let the children experience all the other benefits of PA. This is by far more efficient than reducing risk factors in a few children at high risk. HAPHC also intends to reduce inequality in the health burden regardless of socioeconomic status as we involve whole schools.

It is important that transfer of practices of general attitudes toward PA and learning methods are considered when considering such projects, to achieve the best results for the transfer—and acceptance—of PAAC. By incorporating different countries across Europe, aspects such as different perceptions of PA in different societies and social differences and its impacts on social life and individuals will be studied (64, 65). In some societies, sport and PA can be linked to health and healthy lifestyle, whereas in others it in can be perceived as unnecessary exhaustion and as a potential health hazard.

Attitudes toward, and differences in perception on how the pedagogical process can be best performed in each country will be examined. Some societies link learning with sitting and listening while others emphasize the component of playing and learning *via* doing as an important aspect (66, 67).

Secondary target groups are the teachers and the pan-European school system. Spreading a new type of curriculum that both enhances health and academic performance in children would be a win-win situation for teachers, health authorities and the school-based programs around Europe. Some Nordic countries have more experience in PAAC, while others still have a far way to go, especially in central Europe.

### Selection of Partners

The involved partners in the project are selected on the ground of interest in the field, and the availability to be a participating school. The research collaborators will be selected as they have experienced either data collection and interpretation, development of learning toolkits, organization of workshops or implementation of PAAC in schools. Elementary schools in which the PAAC are to be implemented will be selected based on interest and earlier collaboration with the scientific groups in the respective countries.

Comparing the involved countries in it's current PA status is in itself important, however, establishing an pan-European and culturally transferable teaching toolkits for elementary school teachers and faculties for education with the goal of increasing every countries PA status, health improvement and learning results *via* involvement of these key stakeholders from municipalities, schools and regional administrators, would have in it an health improving capability of tremendous dimensions. These partners will help in establishing good habits for PA on a regular and continuous basis, simultaneously with improved academic performance, in early childhood may have a large impact on future health. Furthermore, an important aspect of including partners from different countries is also that each country has a different educational context, in terms of practices, beliefs toward PA and what constitutes overweight and obesity. For instance, Norway has in the last decade acquired a relatively large knowledge around how to inspire the population, especially children, in increasing the PA level for the benefit of health.

## Possible Limitations

Although the HAPHC project will provide novel information, some limitations have to be considered:

- At date, Austria has confirmed the partnership of three schools, Slovenia and Belgium also of three schools. Unexpected circumstances will be avoided by regular communication and strategy planning, including changing project partner schools. Parents/legal representatives will be informed preliminary about the aims, goals and activities.
- Another limitation may be that outreach activities may not be completed on time. The planned interaction with EU initiatives and involving partners from other regions will strongly support outreach.
- A high risk is that not all the key stakeholders may be engaged by the project in time to carry out appropriate dissemination. It will be ensured that all the stakeholders are engaged and contacted on time. Roles of each partner who should be responsible for which stakeholder have to be identified, although with flexibility.
- A risk is also that a partner drops out, however, all partners have a long tradition of collaboration in different settings, so it is considered to be a low risk. Each partner contributes by being present with his or her specialized competencies and tasks. In case a partner leaves the project, the other partners will cover the lost competences and a substitution will be considered.

## Conclusions

The HAPHC study brings academics from different countries and fields of medical and health sciences as well as education together in a multidisciplinary study to mutual benefit. This study will draw attention from policy makers, media, teachers, parents and children to improve and promote better health among children in Europe. This pan-European partnership—supported by regional and national partners—will go a long way in reducing risk factors for NCDs in children and improve academic achievement.

## Ethics Statement

The studies involving human participants were reviewed and approved by the Medical University of Graz (EK: 33-488 ex 20/2). This study was also approved by the Medical Ethics Committee at Hasselt University (registration no. CME2021/078). Verbal and written informed consent will be obtained from each participant/legal representative prior to being included into the study.

## Author Contributions

All authors listed have made a substantial, direct, and intellectual contribution to the work and approved it for publication.

## Funding

This study receives funding through the ERASMUS + (KA201—Strategic Partnerships for School Education) Program (2020-1-NO01-KA201-076533).

## Conflict of Interest

The authors declare that the research was conducted in the absence of any commercial or financial relationships that could be construed as a potential conflict of interest.

## Publisher's Note

All claims expressed in this article are solely those of the authors and do not necessarily represent those of their affiliated organizations, or those of the publisher, the editors and the reviewers. Any product that may be evaluated in this article, or claim that may be made by its manufacturer, is not guaranteed or endorsed by the publisher.

## Abbreviations

HOPP: Health Oriented Pedagogical Project
MVPA: Moderate to vigorous-intensity physical activity
PA: Physical Activity
PAAC: Physical Activity Across the Curriculum
WHO: World Health Organization.
